# Unilateral Limb Ischemia in a COVID-19 Patient: A Case Report

**DOI:** 10.7759/cureus.34464

**Published:** 2023-01-31

**Authors:** Qusai T Alitter, Ahmad Jarrar, Fahed AlSoud, Islam Gadelmoula

**Affiliations:** 1 Division of Cardiology, Interdisciplinary Stem Cell Institute, University of Miami Leonard M. Miller School of Medicine, Miami, USA; 2 Department of Pulmonary Medicine, Larkin Community Hospital, Hialeah, USA; 3 Department of Internal Medicine, Larkin Community Hospital, South Miami, USA

**Keywords:** venous and arterial thrombosis, vascular ischemia, covid-related hypercoagulability, covid 19 - acute limb ischemia, covid-19 infection

## Abstract

Coronavirus disease 2019 (COVID-19) has been primarily linked to respiratory complications, including acute respiratory distress syndrome (ARDS). However, several systemic manifestations of the disease may also occur. One of the emerging complications that is being increasingly reported in the literature is the hypercoagulable and intense inflammatory state in COVID-19 patients, which leads to venous and/or arterial thrombosis, vasospasm, and ischemia. Despite the recent advances in diagnostic and treatment modalities, the diagnosis and management of vascular ischemia in this patient population remain a challenge, resulting in increased morbidity and mortality. In this case report, we highlight the etiology and potential treatment of limb ischemia in COVID-19 patients.

## Introduction

Coronavirus disease 2019 (COVID-19), caused by the highly virulent coronavirus strain, severe acute respiratory syndrome coronavirus 2 (SARS-CoV-2) has had a devastating impact on the world’s healthcare system and economy, unfortunately resulting in more than 6.6 million deaths worldwide, according to the World Health Organization (WHO). COVID-19 is mainly linked to respiratory illnesses, including acute respiratory distress syndrome (ARDS). However, the infection may also result in an intense inflammatory and pro-thrombotic state, leading to venous and arterial thromboembolism [1], arterial vasospasm, multiple organ failure, and death. Because of the disease's complex pathophysiology, clinicians frequently face difficulties in recognizing the disease's complications and, as a result, delay initiating the proper treatment. In this case report, we highlight and discuss the etiology, early recognition, and potential management of arterial ischemia in COVID-19 patients.

## Case presentation

**Figure 1:**
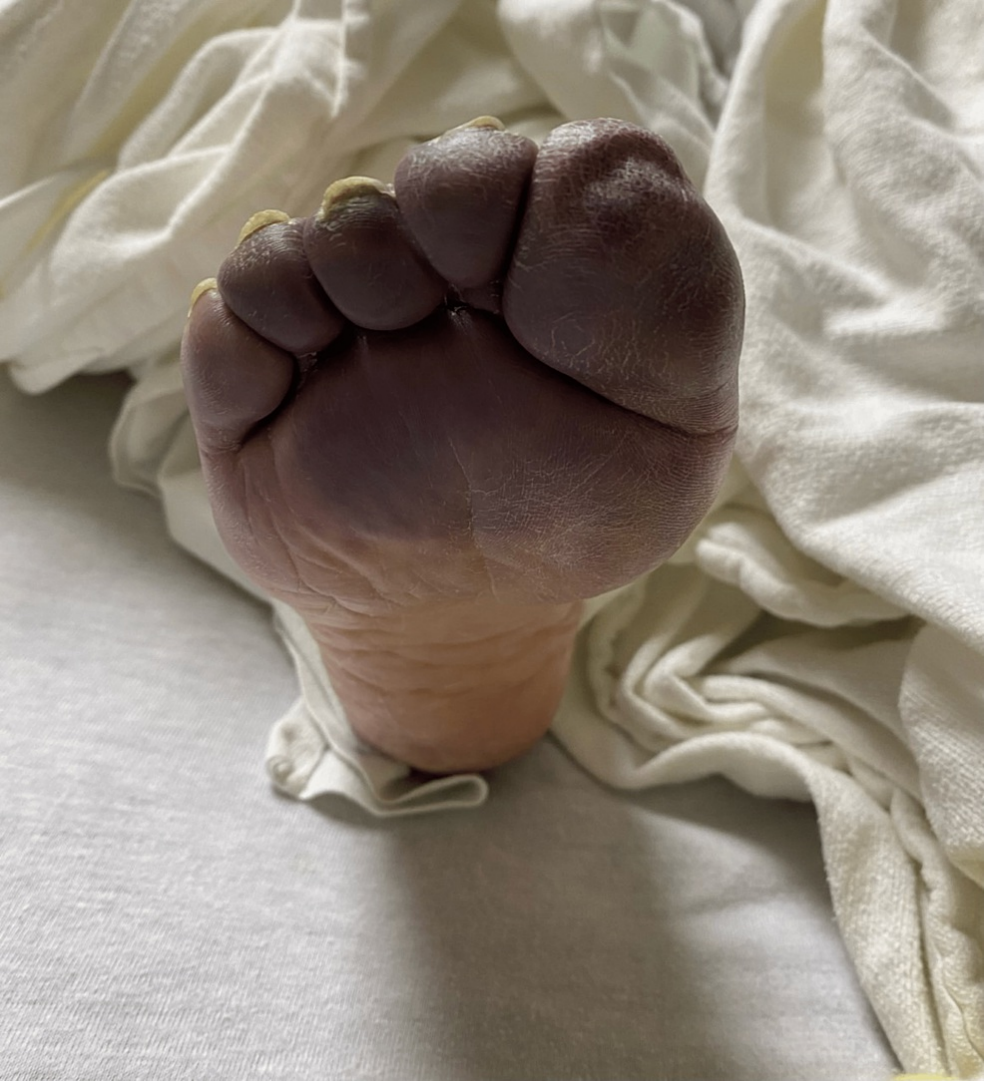
Blue Discoloration of the Right Toes Suggestive of Ischemia

**Figure 2:**
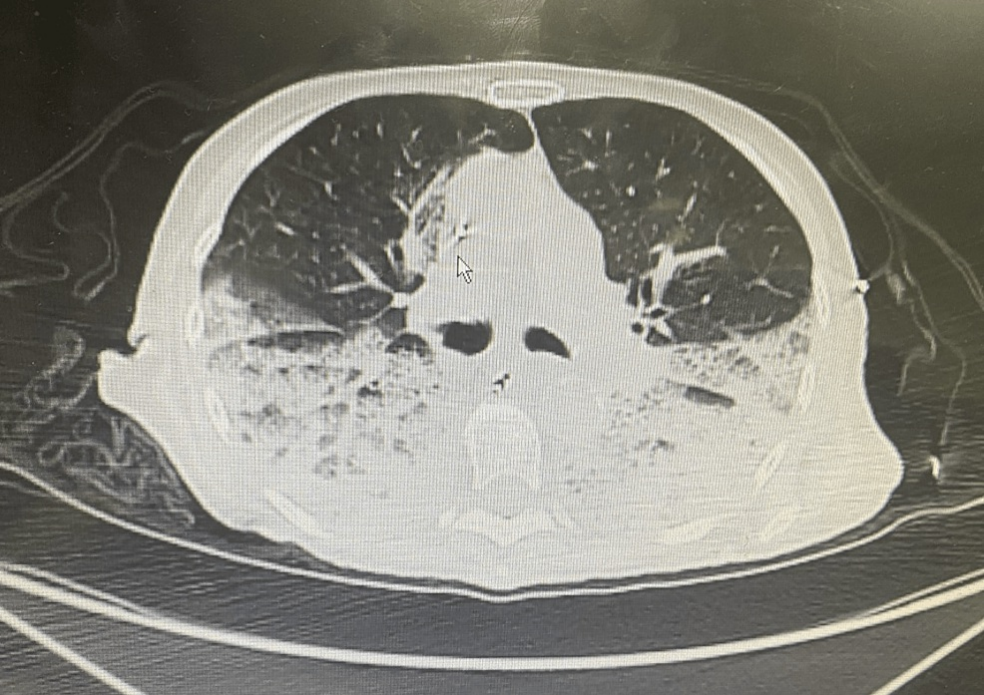
Computed Tomography of the Chest With Evidence of Bilateral Ground Glass Opacities

A 90-year-old female with a past medical history of essential hypertension and non-insulin-dependent diabetes mellitus presented to the emergency department with progressive shortness of breath and right foot pain for three days. According to the patient, the foot pain was triggered by minimal exertion and significantly affected her mobility. She also reports progressive blue discoloration of her right toes, which was not present before. The physical examination was remarkable for bilaterally diminished lung breath sounds and blue discoloration of the right toes (Figure 1). Initial workup in the emergency department (Table 1) was significant for a positive COVID-19 rapid antigen and polymerase chain reaction (PCR) test, a lactic acid level of 4.7 mmol/L, a white blood cell count of 28.48 K/mcl, a D-dimer level of 11.3 μg/ml, mild transaminitis, and an erythrocyte sedimentation rate (ESR) of 56 mm/hr. A computed tomography angiography (CTA) of the chest revealed bilateral ground glass opacities but was negative for pulmonary embolism (Figure 2). An electrocardiogram revealed sinus tachycardia and was negative for acute ischemic changes.

**Table 1:** Initial Workup

Blood Tests and Diagnostic Workup	Result	Reference Range
White Blood Cell Count	28.48 K/mcL	4.00-10.80 K/mcL
Hemoglobin	10.9 g/dl	12-15 g/dl
Platelet Count	161 K/mcL	150-450 K/mcL
Sodium	135 mmol/L	137-146 mmol/L
Potassium	3.9 mmol/L	3.6-5 mmol/L
Carbon Dioxide	17 mmol/L	21-32 mmol/L
Blood Urea Nitrogen	31 mg/dl	8-25 mg/dl
Serum Creatinine	1.2 mg/dl	0.70-1.20 mg/dl
Alanine Transaminase	91 U/L	12-64 U/L
Aspartate Aminotransferase	73 U/L	9-40 U/L
Total Bilirubin	0.9 mg/dl	0.2-1.1 mg/dl
Alkaline Phosphatase	152 U/L	38-127 U/L
C-Reactive Protein	16.8 mg/dl	<0.8 mg/dl
Erythrocyte Sedimentation Rate	56 mm/hr	<15 mm/hr
Influenza A&B	Negative	Negative
COVID-19 PCR	Positive	Negative
Lactic Acid	4.7 nmol/L	0.7-2 nmol/L
D-dimer	11.3 μg/ml	<0.50 μg/ml

Due to 84% oxygen saturation on room air, the patient was started on supplemental oxygen, IV steroids, and remdesivir. A venous ultrasound of the lower extremities was negative for deep vein thrombosis. An arterial ultrasound of the lower extremity was negative for focal occlusion. However, due to the patient's continued complaints of right foot pain, a repeat arterial ultrasound of the right lower extremity was obtained and revealed predominantly monophasic waveforms below the knee, which is consistent with peripheral arterial disease. CTA of the lower extremity showed no evidence of hemodynamically significant occlusion, aneurysm formation, or vascular malformation of the major lower extremity arteries. A repeat D-dimer was also noted to be elevated at 12.6 μg/ml. The patient was started on IV heparin, and a peripheral angiography was planned. However, the patient's respiratory status continued to worsen, requiring intubation. Unfortunately, her condition continued to deteriorate despite multiple therapeutic attempts; she went into asystole and was pronounced dead.

## Discussion

Acute limb ischemia is a medical emergency that can cause significant morbidity and mortality in patients. It is usually caused by a decrease or sudden interruption of limb tissue perfusion, which, if left untreated, leads to tissue necrosis [2]. Arterial vasospasm is a well-known entity, especially in the coronary arteries and the brain [1,3,4]. However, this phenomenon is underappreciated in other vascular systems. COVID-19 has been associated with predominantly respiratory symptoms, but due to a severe pro-inflammatory and immune response, a proportion of patients progress to severe and systemic complications. There are a number of cases of COVID-19-associated acute limb ischemia but most are due to thrombosis due to the disease's prothrombotic state [5].

In a clinical case series conducted by Surya et al., it has been highlighted that the acute COVID-19 infection state has been linked with hypercoagulation, which culminates in the formation of microthrombi known as "immunothrombus" [6]. In a review published by Ortega-Paz et al., the authors investigated the pathophysiological characteristics of cardiovascular system involvement in patients with COVID-19 and ARDS and suggested that the imbalance of angiotensin-converting enzyme and angiotensin-converting enzyme 2 and the subsequent increased levels of angiotensin II, a known potent vasoconstrictor with pro-inflammatory and pro-fibrotic effects, might be responsible for the cardiovascular complications [7].

Acute limb ischemia, even though it is a rare complication of COVID-19, is not only organ-threatening but life-threatening as well. However, due to the complexity of the pathophysiology and clinical presentation of COVID-19, clinicians usually encounter challenges in diagnosing and treating it. Different imaging modalities, including duplex ultrasonography, CT angiography, and magnetic resonance angiography, help establish the diagnosis of limb ischemia. However, digital subtraction arthrography provides the most diagnostic information. Once the diagnosis is established and depending on the severity of the limb ischemia, different treatment options can be initiated, including systemic anticoagulation, intra-arterial thrombolytic therapy, stent implantation, surgical revascularization, and, if the limb is non-viable, amputation [8]. Because of the significant risk of revascularization failure and perioperative death in COVID-19 patients with limb ischemia, post-procedural care and monitoring for complications like compartment syndrome and reperfusion injury are considered crucial parts of the management and can alter the patient's outcome [8].

We believe our patient's limb ischemia is caused by arterial vasospasm rather than microthrombi due to the lack of other organ involvement (other than the lungs and liver), the advanced stage of leg ischemia, the severity of right foot pain the patient was complaining of and the negative workup. Currently, there is no gold standard diagnostic modality for arterial vasospasm; however, one potential treatment option is vasodilators like calcium channel blockers. Still, further studies and research are needed to understand the association between COVID-19 and acute limb ischemia and to establish treatment guidelines for physicians.

## Conclusions

COVID-19 is a complex disease with varying presentations and complications, with acute limb ischemia being a well-known complication of the disease. Acute limb ischemia in this patient population is mainly attributed to the hypercoagulable and pro-inflammatory state of COVID-19; however, arterial vasospasm is another cause of limb ischemia that is underrecognized and scarcely reported in the literature. Because limb ischemia can result in significant mortality and morbidity in COVID-19 patients, early detection and treatment are considered critical steps in management. There are currently no treatment guidelines for arterial vasospasm in this patient population, but one potential option is vasodilators such as calcium channel blockers; however, more research and studies are needed.
